# Predicting the performance of TV series through textual and network analysis: The case of Big Bang Theory

**DOI:** 10.1371/journal.pone.0225306

**Published:** 2019-11-21

**Authors:** Andrea Fronzetti Colladon, Maurizio Naldi

**Affiliations:** 1 Department of Engineering, University of Perugia, Via G. Durant, 93, 06125 Perugia, Italy; 2 Department of Civil Engineering and Computer Science, University of Rome Tor Vergata, Rome, Italy; 3 Department of Law, Economics, Politics and Modern languages, LUMSA University, Rome, Italy; University of Sao Paulo, BRAZIL

## Abstract

TV series represent a growing sector of the entertainment industry. Being able to predict their performance allows a broadcasting network to better focus the high investment needed for their preparation. In this paper, we consider a well known TV series—The Big Bang Theory—to identify factors leading to its success. The factors considered are mostly related to the script, such as the characteristics of dialogues (e.g., length, language complexity, sentiment), while the performance is measured by the reviews submitted by viewers (namely the number of reviews as a measure of popularity and the viewers’ ratings as a measure of appreciation). Through correlation and regression analysis, two sets of predictors are identified respectively for appreciation and popularity. In particular the episode number, the percentage of male viewers, the language complexity and text length emerge as the best predictors for popularity, while again the percentage of male viewers and the language complexity plus the number of we-words and the concentration of dialogues are the best choice for appreciation.

## Introduction

TV series represent a steadily growing business: the number of original scripted TV series in the U.S.A. rose from 210 in 2009 to 487 in 2017 (the data have been taken from the Statista website https://www.statista.com/statistics/444870/scripted-primetime-tv-series-number-usa/, resulting in a CAGR (Compound Annual Growth Rate) of 11.1%.

The associated growing economic importance of TV series and the size of investments needed to fuel them are a prime reason to try to predict their success [1]. A successful predictor of success would allow a broadcasting network to invest on those series whose success we have greater confidence in or even to drive the design of a series so as to make it more successful.

A similar need has been felt for the theatrical movie industry, for which a body of literature has formed in the search for a reliable way of predicting the performance of a movie.

For the case of movies, a number of factors have been proposed to explain their performance. We may classify them according to their nature (intrinsic versus external or social), and their location in time (before the movie release date versus right after that date).

As to the first dichotomy, the class of intrinsic features of the film include its budget, the title, the genre, the director, and the actors. In [2], those factors are called classical. In [3], where all Italian movies in the 1990-1998 period are examined, the economic and artistic success of a movie is related to the economic and artistic success of its director, as well as his/her ties. Such factors are typically known well in advance of the release of the movie.

On the other hand, some external factors may also be considered, such as the sentiment expressed through tweets or posts on forums, or metrics related to the activity on Wikipedia (number of views of the article concerning the movie, number of users, and number of edits). The activity on Wikipedia is considered, e.g., in [2] and [4]. In [5], the success is related to the discussions on forums by taking into account the social network that gets established between the participants in the discussion, where influencers may emerge. Other social factors are the ratings provided by the Motion Picture Association of America (MPAA) [6] or the ratings provided by critics and/or the audience [7]. Additional sources of influence are the references to the movie, exchanged through tweets [8] or on blogs [9]. Those data may be known slightly in advance of the movie release date, for example as rumours accumulate or from people who have had a chance to view some trailers. But they may also explode right after the release of the movie, when all the first viewers run to share their impressions.

A special approach is that adopted in [10], where neural networks are employed as a prediction/explanation tool (rather than regression analysis as in all the other papers) and some additional factors are considered, such as the presence of technical effects, whether the movie is a sequel, and the number of screens.

In all cases the measure of performance is represented by the revenues.

Though an adequate body of literature is present for movies, TV series are still a rather unexplored territory. Results obtained for movies cannot be straightforwardly applied to TV series, due to their different nature. TV series extend over a long period of time, so that they can have an evolution. Their appreciation among the audience may change, reflecting a change in the taste of the public. But their plot and characters may also change by the choice of the TV series designers, often to meet the audience’s preferences or to change the target audience. In addition, revenues cannot be directly associated to the TV series as they are for a movie, so that they cannot be easily adopted as a measure of success.

Some studies have however investigated some issues concerning the success of TV shows. Kennedy found that broadcasters’ strategies tend to imitate each other, but such a herding behaviour is not paying, since imitative introductions underperform differentiated intoductions [11]. Differentiation, however, becomes increasingly difficult as the offer increases, so that niches become saturated and the audience becomes satiated with the current offer, as found out through the analysis of the survival rates of television series aired in the United States from 1946 to 2003 [12]. Another factor for success was found to be related to the name of the TV programme [13]. These studies have focussed on the ecosystem of TV programming, rather than examining the actual plot structure and contents of the series.

At any rate, predicting the success of TV shows has always been a prone-to-failure task, as the case of *Seinfeld* shows, where success came after a change of schedule and four years of poor performance [14].

In this paper, we deal with the problem of identifying the factors of success for a TV series. As an initial step, we focus on a specific TV series: The Big Bang Theory. This series can boast a wide array of awards and has been distributed over many countries. As hinted before, we move away from many approaches adopted for theatrical movies as well as early attempts on TV series. We have focussed on the contribution to performance provided by the intrinsic contents of the TV series as represented by its script, using text analysis and social network analysis to describe the interactions between its characters. In particular the following original choices have been made:

- we do not use revenues as a measure of performance, but adopt the ratings assigned by viewers as well as the number of reviewers (voters);
- we consider the social network that builds among the characters of the TV series through the dialogues; and
- we use text mining to extract metrics describing the complexity of the language and the sentiment expressed in dialogues.

As to the first item, it is to be noted that the opinions of viewers are employed in some papers on movies as predictors (though they typically occur *after* a movie is released), as in [15] for the role of special viewers such as the critics, or [16] for the reviews delivered by general viewers on the NAVER movie review platform. Here we use it instead as the variable to be predicted.

If we understand which features account for the performance of the series, the series designers can push those features at the expense of others that are less relevant or even hurting the popularity of the series itself. Though our analysis encompasses the whole length of the Big Bang Theory, the investigation approach can be applied as the series progresses to extract information useful for the episodes to be planned.

The paper is organized as follows. In “The data set” section, we describe the data set that we have employed in our analysis, i.e., the script of the Big Bang Theory, including two metrics of performance. In the “Predictors of Appreciation and Popularity” section, we define the predictors we have chosen. Finally, in the “Optimal choice of predictors” section, we apply correlation and regression analysis to perform a screening on our list of potential predictors and come out with the most significant ones.

## The data set

We have employed two data sets, concerning respectively the dialogues taking place between the characters in that Big Bang Theory episode and the rating obtained by that episode. In this section we describe those data sets.

The Big Bang Theory is an American television sitcom, premiered on CBS in 2007. It has now reached its twelfth season. After a slow start (ranking 68th in the first season and 40th in its second one), it ranked as CBS’s highest-rated show in that evening on its first episode in the third season.

We have considered all the episodes from the initial one of Series 1 to the 24th episode of Series 10 (https://bigbangtrans.wordpress.com), for a total of 232 episodes.

**Fig 1:**
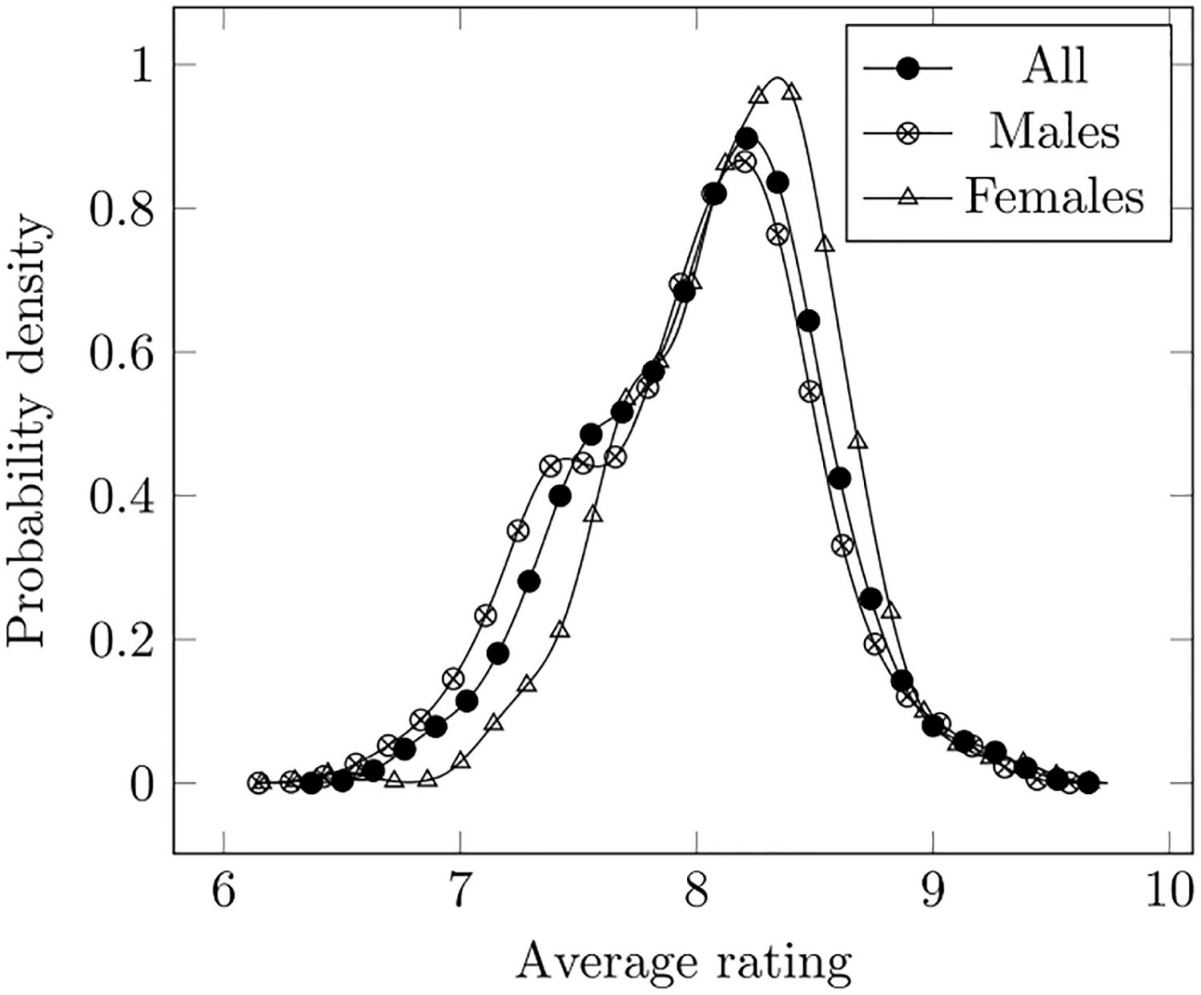
Distribution of average ratings per episode.

The ratings (and the voters) for each episode have been retrieved as posted on the Internet Movie Database (IMDb). The sample of posts is therefore contributed by IMDb-registered people, who might not reflect the whole population of viewers, as is the case in any non-controlled experiment. All the reviews are available on the IMDb page (https://www.imdb.com/title/tt0898266/ by clicking on the “Review” tab. No signing in or account set-up was needed, so that anyone can access them without registration. We manually copied the reviews on IMDb, and did not use data extraction tools which are prohibited by IMDb’s Conditions of Use (https://www.imdb.com/conditions). The demographics information (including gender and age) were retrieved on a summary box made available by IMDb for each episode (see, e.g., https://www.imdb.com/title/tt3603346/ratings?demo=imdb_users). Ratings are expressed on a scale from 1 to 10. In Fig 1 we show the distribution of average ratings (i.e., the average rating for each episode is considered) observed on the 232 episodes, as estimated through a Gaussian kernel approach [17]. As can be seen, the range actually swept is roughly between 7 and 9 and women provide slightly larger scores (their distribution lies more on the right).

While ratings express the audience appreciation for a TV series episode, the number of voters, on the other hand, can be considered as a proxy of its popularity. The idea is that the higher the number of people who vote for an episode, the larger its audience.

**Fig 2:**
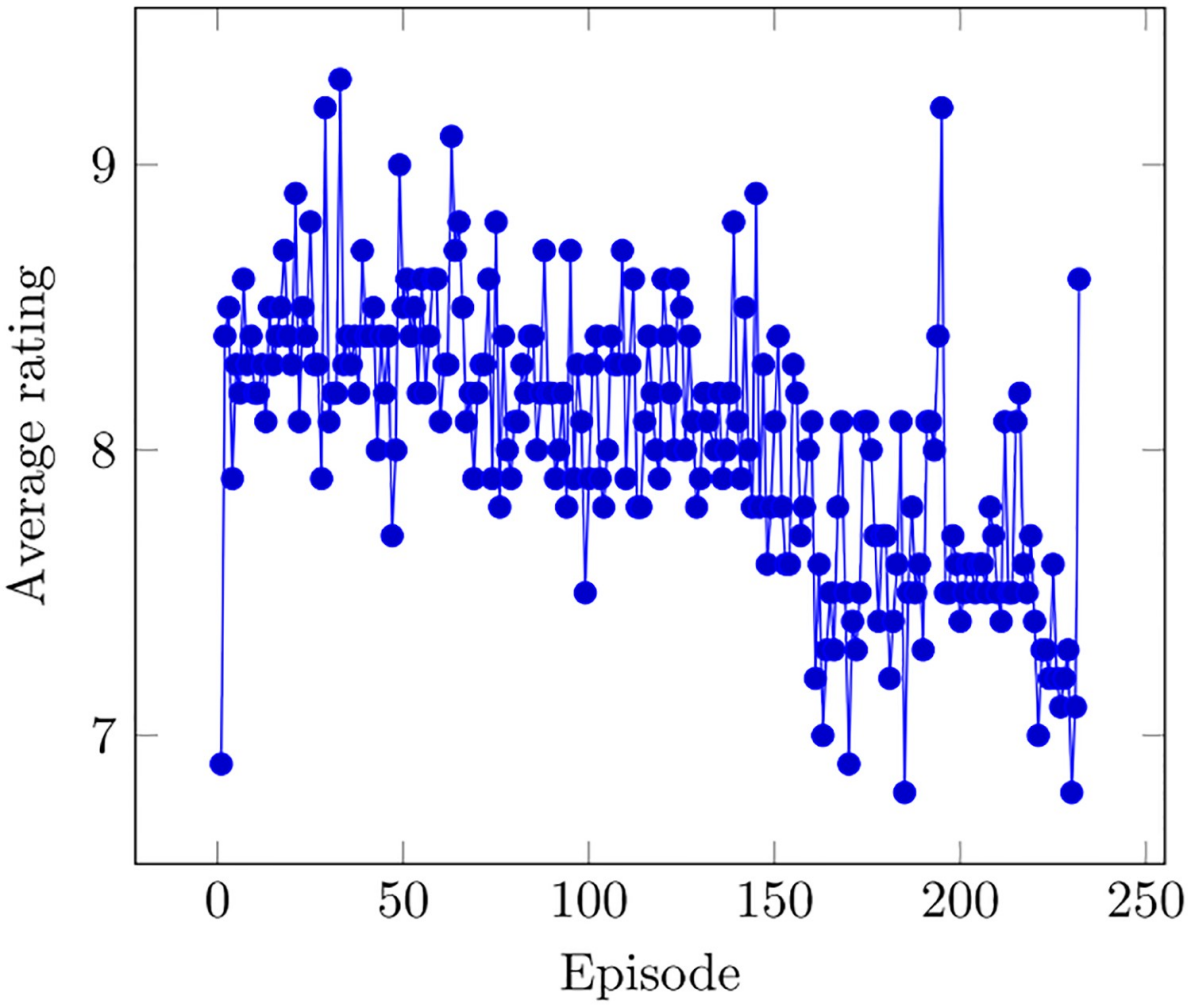
Time evolution of average ratings.

**Fig 3:**
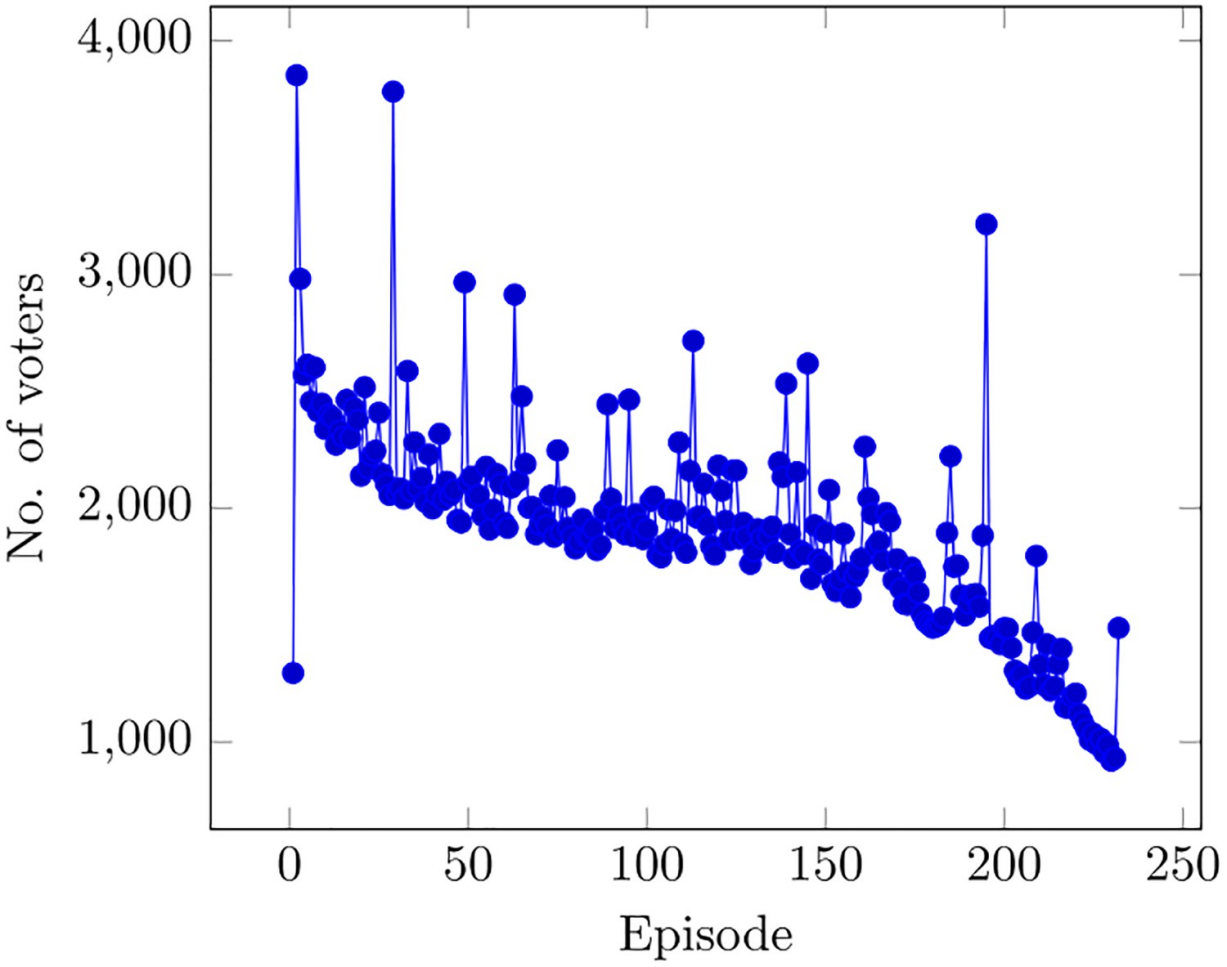
Time evolution of voters.

In the end, we use therefore two metrics of performance: viewers’ ratings as a measure of appreciation, and the number of voters as a measure of popularity. The time series of average ratings (again, the average rating for each episode is considered) and voters are shown respectively in Figs 2 and 3. A negative trend can be observed in both.

## Predictors of appreciation and popularity

As reported in the Introduction, many authors have provided their choice of metrics to predict the success of a movie. We step aside from the approaches taken so far for movies, by adopting predictors related to the script of the series. The most important quality of these predictors is that they are known by the TV series designers largely in advance of the series release. In addition, they can easily be acted upon (certainly more easily than changing other intrinsic factors suggested in the literature, such as the actors or the director). In this section, we describe each predictor.

As potential predictors of performance, we employ a number of variables, borrowed from the textual analysis of the episode’s content and the analysis of the social network existing among the characters in the episode. What distinguishes this choice from the selection of predictors proposed in the literature is that ours considers predictors extracted from the episode contents.

Our selection includes metrics describing the following characteristics:

1. Season;
2. Episode number within the season;
3. Text length;
4. Complexity of the text;
5. Language sentiment;
6. Use of function words;
7. Concentration of dialogues;
8. Gender of voters.

We can roughly divide these indicators in four groups, describing respectively the temporal location of the episode (Metrics 1 and 2), the text characteristics (Metrics 3 through 6), the relationship among the characters (Metric 7), and the characteristics of the voters (Metric 8).

The first two metrics are quite straightforward and allow us to examine any possible time dependency of audience appreciation, e.g., if the ratings increase over time because positive reactions from the audience build up and spread attracting new viewers. On the other hand, it could also happen that the popularity is high at the start of the series because of expectations, but then decreases because those expectations are not fulfilled. For example, in [18], it was noted that the popularity of TV shows decreases over time.

**Fig 4:**
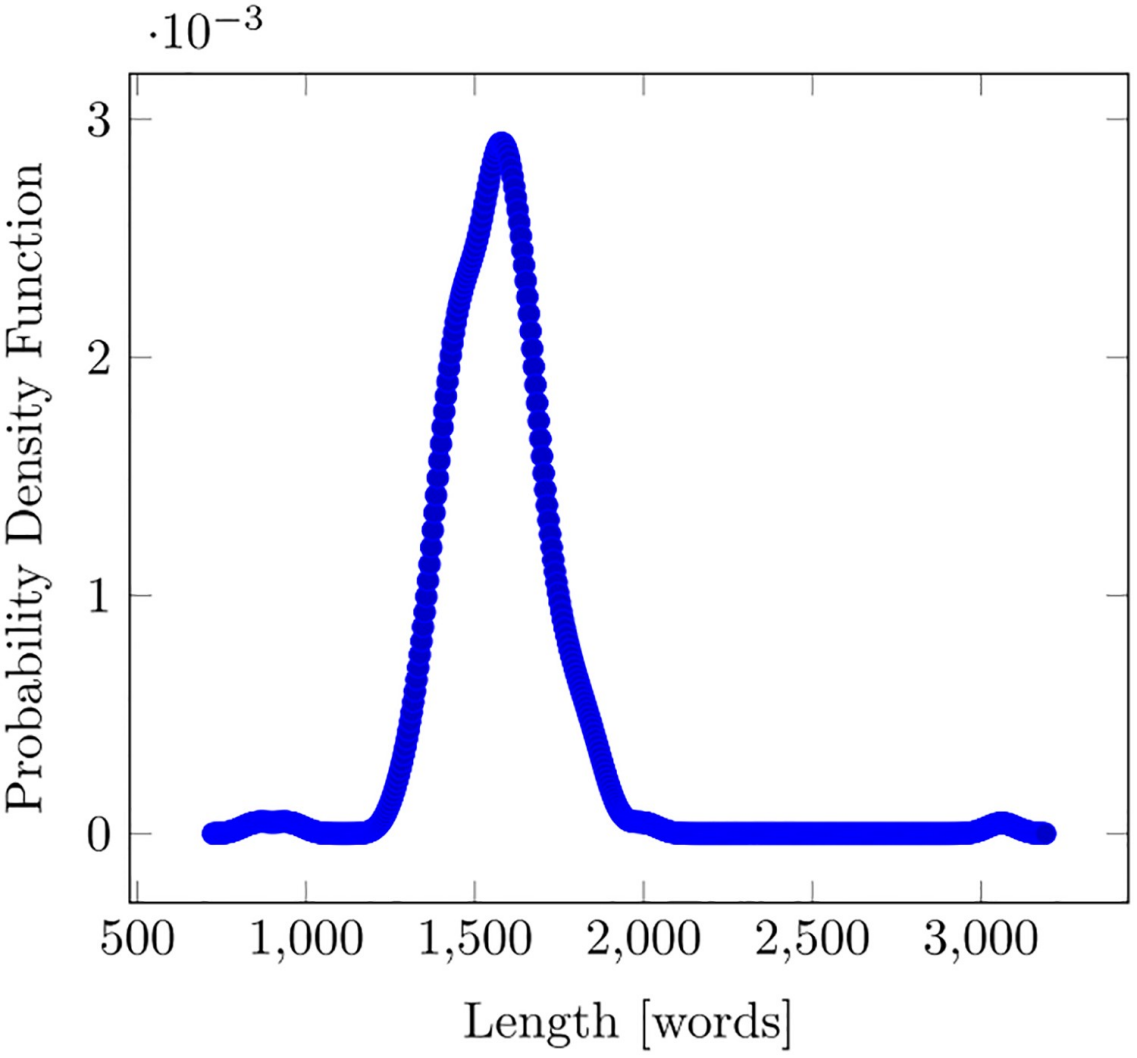
Distribution of episode length in words.

The text length is represented by the overall number of words appearing in the dialogues over an episode. The duration of each episode is rather standard, being constrained by the typical schedule of TV broadcasts, which are arranged to have programmes starting either on the hour or at subsequent quarters (e.g., at 10.00 or 10.15, 10.30, and 10.45), so that most programmes conform to a duration of multiples of 15 minutes. However, we can show that the textual length is far from being equal for all episodes. In Fig 4, we see that the distribution of length is markedly non uniform. It is also quite perfectly symmetric: its mean value and its median value are practically equal (1566.6 words versus 1566). The coefficient of variation (i.e., the ratio of the standard deviation to the mean value) is 0.11: the dispersion is small but enough to exhibit differences between the episodes. The episode length, expressed in words, is therefore a suitable variable to characterize each episode.

As to the complexity of the text, we have adopted a very simple indicator. We assume that the complexity of the text is represented by the complexity of the vocabulary, and this in turn is described by the variance of the frequency of the words used in the text. We consider the rank-frequency distribution of frequencies, where *f*_(1)_ represents the number of occurrences of the most frequent word (*f*_(2)_ that of the second most frequent and so on). If we denote by *n* the number of distinct words appearing in the text, the average frequency is

$$\begin{array}{c} \bar{f}=\frac{\sum_{i=1}^{n}f_{\left(i\right)}}{n} \end{array}$$ (1)

and the variance is instead

$$\begin{array}{c} \sigma_{f}^{2}=\frac{\sum_{i=1}^{n}(f_{\left(i\right)}-\bar{f}{)}^{2}}{n}. \end{array}$$ (2)

The variance in Eq (2) is the indicator we have chosen to represent the complexity. The rationale for this notion of complexity is that the more the language converges towards the use of a smaller and more uniform set of words, the lower its complexity [19, 20]. In general, we consider a word more complex when it appears more rarely in a specific context and not when just rarer in general. This latter notion is in line with the findings of [21]: words of higher frequencies require less extra effort when they are retrieved from the reader’s mental lexicon.

We can get a feeling of the characteristics of the frequency variance indicator by observing what happens for a well-known rank-frequency distribution in statistical text analysis: the Zipf law [22]. In its original formulation in [23], the Zipf law states that the frequency of a word in a text is inversely proportional to its rank. The generalized version states instead that the frequency is inversely proportional to a power of its rank:

$$\begin{array}{c} f_{\left(i\right)}=\frac{k}{i^{\alpha }}\;i=1,2,\ldots \end{array}$$ (3)

where *k* is the number of occurrences of the most frequent word. The value of the Zipf exponent *α* varies over national languages and specialized domains. In [24] it has been reported that *α* = 1.13 for the American National Corpus, and that it can vary from 0.51 to 1.88 depending on the national language (just out of curiosity, the smallest value pertains to Maori and the largest one to Russian).

**Fig 5:**
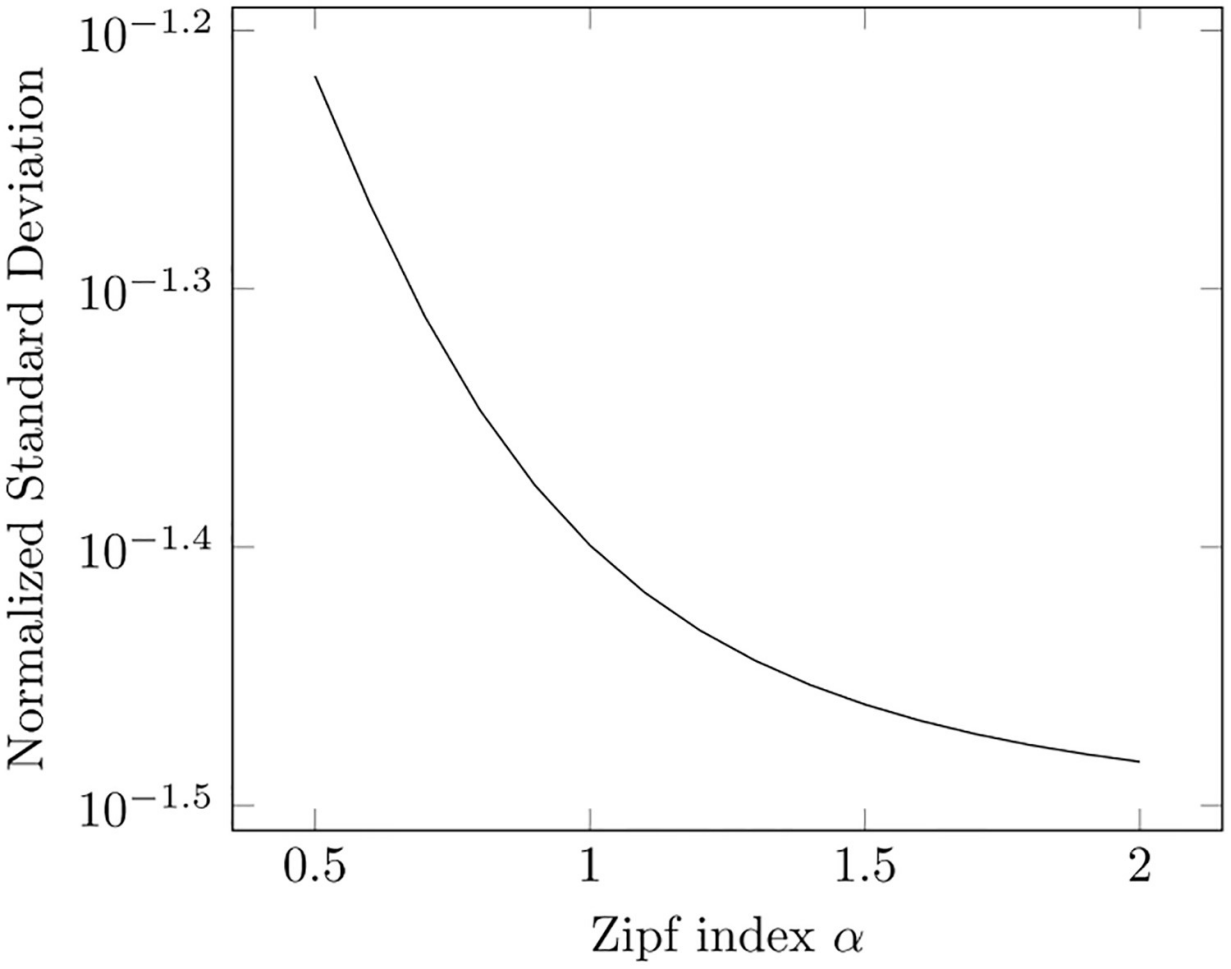
Standard deviation of frequency under Zipf law.

Over a finite set of distinct words, the use of Eq (3) would require the use of the truncated Riemann Zeta function, for whose sum no closed form exists. We may resort to the trapezoidal approximation proposed in [25] and obtain an approximate closed form, but we prefer showing the resulting normalized standard deviation of frequencies *σ*_f_/*k* (i.e., divided by *k*, the number of occurrences of the most frequent words) in Fig 5 for the theoretical case of Zipf law. We see that the standardized deviation (i.e., the proxy for complexity)) decays with the Zipf index much faster than exponentially.

We recall however that we measure complexity over the specific corpus represented by the episodes of BBT rather than over a very large corpus as that implied by the use of Zipf law. We do not adopt a dictionary-based approach, since it would leave out words or names (e.g., the names of characters) that are absent in general dictionaries, but still very commonly used in TV transcripts. For example, while a dictionary-based approach might classify the word “Sheldon” as very rare, we know that this is not true for the case of BBT. On the other side, we are aware that the example represented by Zipf law is reported just as a reference case, without expectation of finding a similar behaviour.

Language sentiment has been measured using the machine learning algorithm included in the Condor software for semantic and social network analysis [26] (previously known as TecFlow [27]), which adopts a naive Bayes classifier [28]. The sentiment score *LS* here describes the positivity or negativity of the language used in an episode. It varies in the [0, 1] interval, ranging from a text conveying only negative feelings (*LS* = 0) to its opposite of a fully positive text (*LS* = 1). The half-range score *LS* = 0.5 indicates a perfectly neutral sentiment. The algorithm has been applied, e.g., to the analysis of trends in a social network [29] and the analysis of Twitter data by Brönnimann [30, 31].

The next indicators in our list concern the use of function words by the series characters. Function words are mainly pronouns, articles, and prepositions (plus a handful of minor words) that account for less than 0.1% of our vocabulary but make up almost 60% of the words we use. As claimed in the seminal book by Pennebaker [32], the use of those words give us an insight into the personality and social connections of people [33]. We wish to see if the revealing properties of function words about the personality and interactions of the series characters have an influence on the appreciation of the series by the audience. For that purpose, we have created two variables, which count respectively the number of *I*- and *We*-words in the text (e.g. “I”, “me”, “my”, and “mine” versus “we”, “us”, “our”, and “ours”). According to Pennebaker a person with higher status in a dyadic conversation within a group uses fewer I-words and more we-words [32, 34]. Similar findings have been reported in studies concerning the online collaboration among professionals of different background [35], and even in Saddam Hussein’s circle of collaborators [36]. The number of I-words and we-words would therefore allow us to see if the appreciation of the series is somewhat linked to the presence of characters possessing these traits.

**Fig 6:**
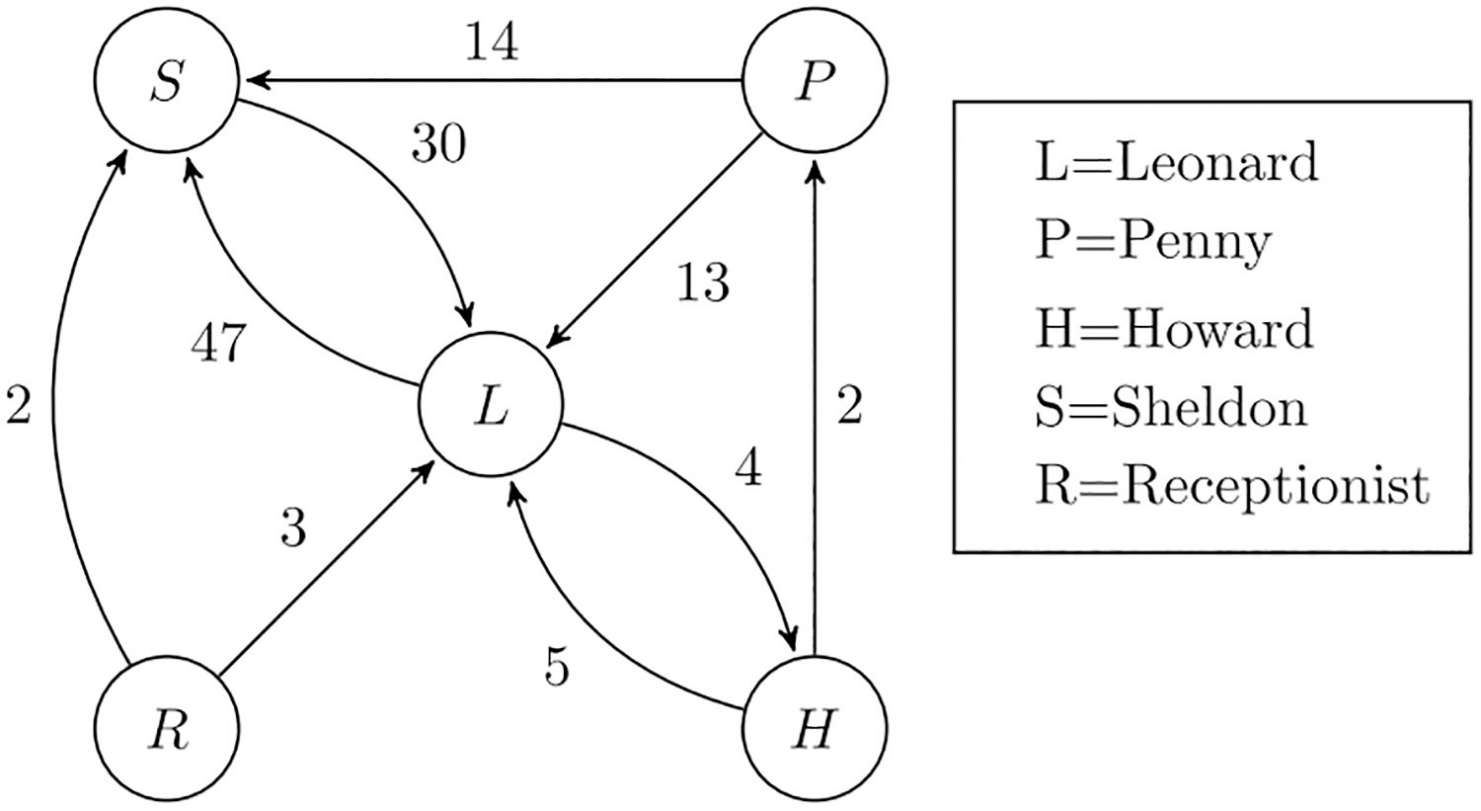
Dialogue graph for Episode 1 of Season 1.

We now turn to an indicator we choose to describe the interactions among the actors playing in each episode. For that purpose we build a social network among the TV series characters. That network is therefore represented by a graph with a number of nodes equal to the number of characters. We have a link from node *i* to node *j* if the character associated to node *i* speaks to that associated to node *j* as embodied by the presence of at least a line of character *i* followed by a line of character *j*, irrespective of the actual number of words used by character *i*, having removed the lines made of simple fillers. Links are weighted, with the weight represented by the number of times character *i* speaks to character*j*. Summing up, the resulting graph is directed and weighted. In Fig 6 we report the resulting graph for Episode 1 of the first season (in order not to garble the graph, we have not reported the links concerning single instances, i.e. when character A speaks just once to character B). In that graph, for example, we can read that in that episode Penny speaks 14 times to Sheldon, i.e., Penny has 14 lines in the script where she’s followed by a reply by Sheldon.

After building the dialogue graph, we wish to indicate how one or more characters may dominate the episode. In other words, we wish to build a dominance index. For that purpose we adapt an index borrowed from the field of industrial economics, named the Hirschman-Herfindahl Index (or HHI, for short) [37–39]. In order to see if an industry is concentrated in the hands of a few companies, the HHI is defined as the sum of the squared market shares of all the companies in the market. In our case, we similarly define HHI as our dominance index by considering the number of times a character speaks to any other character (i.e., the number of lines of that character, in the theatre jargon). Using the number of lines as a measure of relevance, rather than the number of words. is supported, e.g., in simple analyses of the roles of characters in TV series. That is reported, e.g., on the page https://yashuseth.blog/2017/12/29/data-analysis-lead-character-of-friends-data-science/ or on the page https://www.reddit.com/r/gameofthrones/comments/4s2n6z/tv_characters_by_\number_of_spoken_lines_in_game/.

The equivalent of the *market share* in this context is therefore the fraction of lines of each character with respect to the overall number of lines in the episode. Turning back to the example of Fig 6, we can build a dialogue matrix, which reports the weights of each edge. Considering a mapping between character names and indices as that suggested by the legend in Fig 6 (i.e., 1 for Leonard, 2 for Penny, and so on), we obtain the following dialogue matrix **D**

$$\begin{array}{c} D=(\begin{array}{ccccc} 0 & 0 & 4 & 47 & 0\\ 13 & 0 & 0 & 14 & 0\\ 5 & 2 & 0 & 0 & 0\\ 30 & 0 & 0 & 0 & 0\\ 3 & 0 & 0 & 2 & 0 \end{array}) \end{array}$$ (4)

By indicating the element of matrix **D** of place (*i*, *j*) by *d*_*ij*_, for an episode in which *n* character appear, the HHI is

$$\begin{array}{c} \text{HHI}=\frac{\sum_{i=1}^{n}{\left(\sum_{j=1}^{n}d_{ij}\right)}^{2}}{{\left(\sum_{i=1}^{n}\sum_{j=1}^{n}d_{ij}\right)}^{2}} \end{array}$$ (5)

The lines for the group of 5 characters are reported in the following vector

$$\begin{array}{c} \left(\begin{array}{c} 51\\ 27\\ 7\\ 30\\ 5 \end{array}\right) \end{array}$$

The overall number of lines is 120, so that the resulting dominance index for that episode is HHI ≃ 0.299.

Finally, the last metric is the percentage of male voters, which accounts for the possible presence of gender-related preferences. Though the gender is not a feature that we can act upon and therefore cannot be employed to increase the appreciation of a series (i.e., it is not a series design parameter), it can be exploited to orientate other choices, e.g., the commercials.

## Optimal choice of predictors

After introducing a set of metrics for potential predictors of appreciation and popularity, we now turn to their analysis. In this section, we report the results obtained for the BBT series. The tools we adopt are both correlation and regression.

A first step in the analysis of the possible determinants of appreciation and popularity of the Big Bang Theory episodes was to look for their significant correlations with text characteristics of their scripts. As a correlation metric, we have adopted Spearman’s rank-order correlation coefficient *ρ* to determine if a monotonic relationship exists between the variables of interest.

In particular, we test such a monotonic relationship between either the episode appreciation (embodied by the rating) or the popularity (embodied by the number of voters) and each of the predictors described in Section “Predictors of Appreciation and Popularity”.

To define the coefficient *ρ* we consider two generic variables *X* and *Y* whose correlation we wish to assess. In our case *Y* is either the rating or the number of voters, and *X* is any of the predictors. After sorting the values obtained for each variables over the *n* episodes, we can observe the ranks of each episode $R_{i}^{\left(X\right)}$ and $R_{i}^{\left(Y\right)}$, *i* = 1, 2, …, *n*. For example, if we are considering the correlation between popularity and language sentiment, and episode 7 ranks third as popularity and fifth as language sentiment, we have $R_{7}^{\left(X\right)}=5$ and $R_{7}^{\left(Y\right)}=3$. Spearman’s rank-order correlation coefficient is then defined as [40]

$$\begin{array}{c} \rho =1-\frac{6\sum_{i=1}^{n}{\left(R_{i}^{\left(X\right)}-R_{i}^{\left(Y\right)}\right)}^{2}}{n(n^{2}-1)} \end{array}$$ (6)

Tables 1 and 2 respectively report the values of *ρ* obtained for appreciation (rating) and popularity (voters). The presence of asterisks indicates the results of testing the null hypothesis that the variable are not correlated, with a double asterisk meaning that the null hypothesis is rejected with a 1% significance level, and a single asterisk corresponding to a rejection with a 5% significance level. As can be seen, with a single exception, all the results show a significant correlation.

**Table 1:** Spearman correlation coefficient for rating (†*p* < 0.001; ⊗*p* < 0.01; *p < 0.05).

Predictor	Spearman *ρ*
Voters	0.783^⊗^
Percentage of males	-0.577^⊗^
Complexity	0.316^⊗^
I-words	-0.302^⊗^
We-words	-0.277^⊗^
HHI Concentration Index	0.258^⊗^
Sentiment	-0.165^⊗^
No. of words	0.158*
Episode	-0.011

**Table 2:** Spearman correlation coefficient for voters (†*p* < 0.001; ⊗*p* < 0.01; *p < 0.05).

Predictor	Spearman *ρ*
Rating	0.783^⊗^
Percentage of males	-0.601^⊗^
Complexity	0.484^⊗^
HHI Concentration Index	0.432^⊗^
Episode	-0.379^⊗^
I-words	-0.338^⊗^
No. of words	0.308*
Sentiment	-0.254^⊗^
We-words	-0.245^⊗^

As the table shows, all our variables significantly correlate with appreciation and popularity of BBT episodes. In general, it seems that more articulated episodes are more popular and obtain higher ratings, as suggested by the significant and positive correlations with number of words and complexity. HHI is also positively related with both the dependent variables. Surprisingly, language sentiment is negatively associated with rating and popularity, suggesting that the audience may appreciate more a less positive (maybe conflicting) language. Similarly, we notice a negative association of the use of I- and we-words with ratings and popularity. Therefore, the audience seems to prefer impersonal statements over a personalized language. As to the other variables, we see that the first episodes of a season are more popular than the subsequent ones, getting a higher number of votes. Moreover, there is a negative association of the rating variable with the percentage of male voters, i.e., female voters provide higher ratings. We can therefore guess that the current features of the series are more appreciated by women than by men. Though we definitely cannot use the gender as a predictor of success, since it is not a variable that we can act upon, we can envisage to use that information to orientate commercials: if women like the series more, and will probably keep on watching it, commercials orientated to a female audience should be preferred. Going further, we can envisage to track correlation between ratings and features, and ratings and gender to understand which features are most liked by each gender.

In order to better test the significance of our predictors, we implemented the multilevel regression models with random intercepts (see [41] for a description of multilevel regression models). We consider a model with two levels: episodes (Level 1) and seasons (Level 2), with the episodes levels nested within the seasons level. We first tested the contribution of each block of predictors, starting with the empty model (Model 1), considering models with single predictors (Models 2 through 7, and Model 8 for the set of main characters), and then building the full model with and without the gender feature (Models 9 and 10). We have also considered the presence of characters in each episode, in addition to the features defined in the previous section. In Table 3, we show the model parameters when the dependent variable is the Rating. We see that the inclusion of gender (Model 9) leads to the largest variance reduction: though we state once and again that it cannot be used as a predictor, these results show the relevance of gender of voters in the rating scored by the series.

**Table 3:** Slopes and variances in the multilevel model for rating (†*p* < 0.001; ⊗*p* < 0.01; *p < 0.05).

Predictor	Model									
	1	2	3	4	5	6	7	8	9	10
Episode		0.003							0.0042	0.0028
Male percent.			−14.0^†^						−15.1^†^	
Complexity				0.0036					0.0057*	0.0066
Sentiment				-0.51					-1.43	-1.11
No. of words					-0.00003				0.00008	0.00006
I-words						-0.0012			-0.0015	-0.0018
We-words						-0.0038			−0.0085^†^	−0.0065^⊗^
HHI							-0.84		−1.47^⊗^	−1.48*
Amy								0.0053	-0.001	0.0043
Bernadette								-0.0034	-0.0003	-0.0044
Emily								-0.0005	0.0037	-0.0005
Howard								0.0005	0.0015	0.0006
Leonard								0.001	0.0019	0.0014
Leslie Winkle								-0.0066	-0.0061	-0.0099
Penny								-0.0006	-0.0013	-0.0005
Raj								-0.0028	0.0028	-0.0015
Sheldon								-0.0014	0.001	0.0004
Stuart								-0.0103	−0.0148*	−0.016*
Intercept	8.11^†^	8.077^†^	19.79^†^	8.30^†^	8.157^†^	8.368^†^	8.282^†^	8.157^†^	21.772^†^	9.105^†^
L2 Variance	0.0786	0.0793	0.0411	0.0702	0.0794	0.0617	0.0934	0.0825	0.0185	0.0544
L1 Variance	0.105	0.105	0.085	0.104	0.105	0.104	0.103	0.102	0.076	0.095

**Fig 7:**
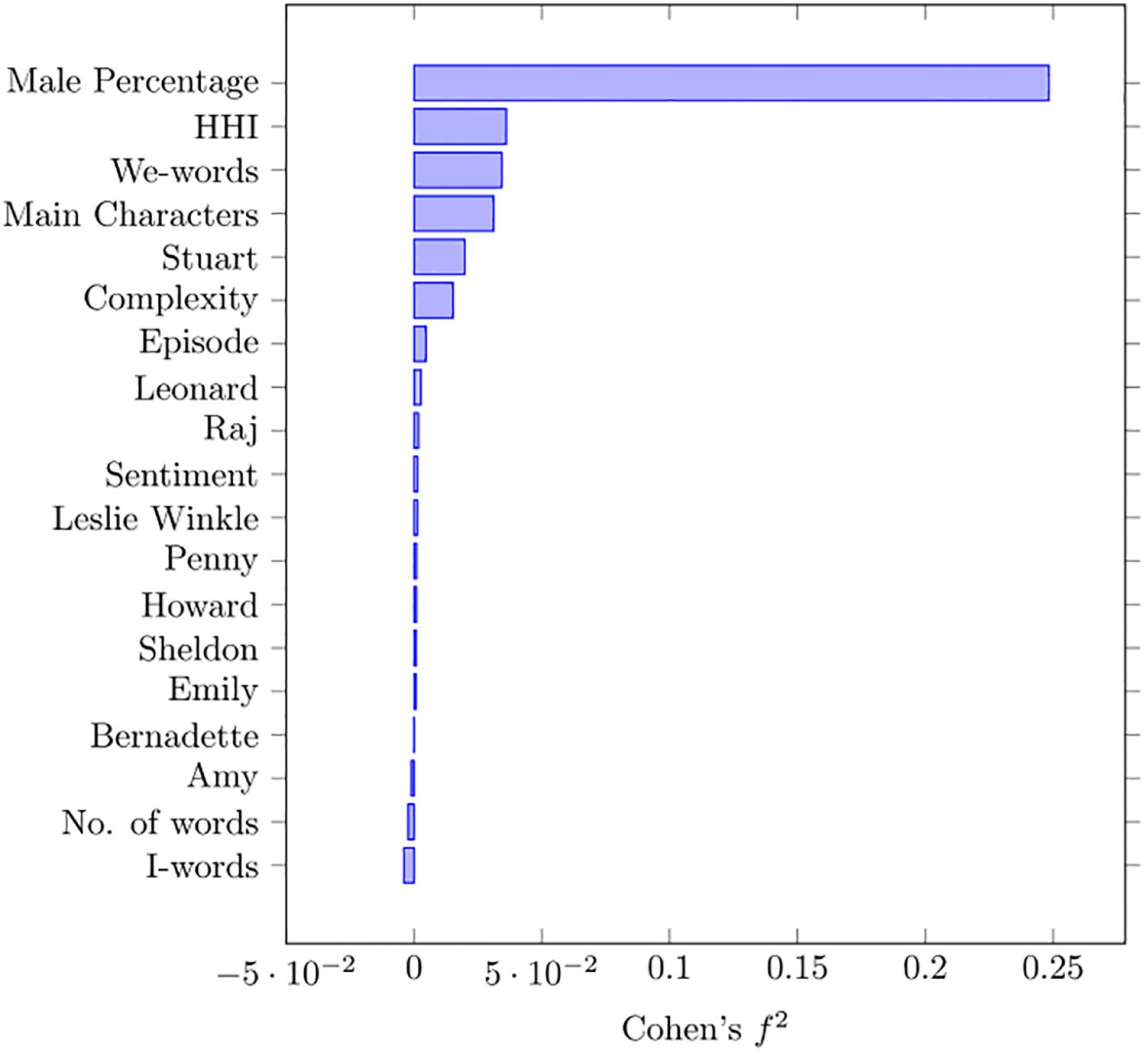
Cohen’s *f*^2^ for ratings.

In order to assess the importance of those predictors, we resort to Cohen’s *f*^2^ effect size, which measured the relative reduction in the variance when we remove a single predictor from the full model, i.e.

$$\begin{array}{c} f_{a}^{2}=\frac{R_{0}^{2}-R_{a}^{2}}{1-R_{0}^{2}}, \end{array}$$ (7)

where $R_{0}^{2}$ is the R squared pertaining to the full model, and $R_{a}^{2}$ is what we obtain after removing the predictor *a*. In Fig 7, we see that the largest effect is due to the percentage of male viewers. The best predictors are then:

- Male percentage;
- HHI;
- We-words;
- Stuart;
- Complexity.

As Table 3 shows, the percentage of male voters can significantly affect the ratings: having more males translates into lower ratings on the average. Also, the presence of Stuart reduces the appreciation of the series, but the effects of any other character is negligible. These results partially confirm those obtained through the correlation analysis reported in Table 1, showing that episodes with lower sentiment and a more impersonal writing style get higher ratings. On the other hand, the sign of the HHI coefficient is negative, suggesting that the audience appreciates more decentralized interactions, with more heterogeneous patterns. We could call this a more *democratic* participation of the characters included in the episodes.

If we want a parsimonious model, we can keep those predictors signalled by Cohen’s *f*^2^. The resulting coefficients are shown in Table 4. We see that the L1 variance is very close to that obtained with the full model inclusive of gender (the price paid for parsimony is a negligible 1.4% increase in L1 variance, while the full model exclusive gender would lead to a quite larger 24.8% increase) and can therefore be considered as an excellent approximation.

**Table 4:** Slopes and variances in the parsimonious model for rating (†*p* < 0.001; ⊗*p* < 0.01; *p < 0.05).

Predictor	Weight
Male percentage	−14.49^†^
Complexity	0.00579
We-words	−0.0065^⊗^
HHI	−1.463^⊗^
Stuart	−0.014*
Intercept	20.477^†^
L2 Variance	0.030
L1 Variance	0.077

We can also examine if the most appreciated episodes (i.e., those with the highest average rating) exhibit significant differences in their features with respect to the other episodes: if a significant difference exists, those features may be considered as being representative of well performing episodes. For this purpose, we have compared the Top 10% episodes with the remaining 90% i.e., the bottom 90%. The comparison has been carried out by applying a two-samples t-test (Test 11 of [40]), after a preliminary Levene’s test [42] on the equality of variances in the two groups (Top 10% vs Others). For that purpose we computed separately the arithmetic averages of each predictor respectively over the episodes that got the Top 10% scores and over those that got scores in the bottom 90% range, and input them to a t-test, where the null hypothesis is that the two groups come from the same population and the difference between their averages is not statistically significant. We report the main results in Table 5, i.e. the average values of each predictor for the two groups (episodes with rating in the Top 10% and bottom 90% respectively) for each predictor and the p-values obtained from the test.

**Table 5:** T-test results for top 10% versus bottom 90% comparison.

Predictor	Average (Top 10%)	Average (Bottom 90%)	p-value
Episode	12.77	12.01	0.593
Male percentage	0.822	0.833	0.000
Sentiment	0.550	0.553	0.328
No. of words	1546	1569	0.534
Complexity	31.23	26.02	0.015
HHI	0.222	0.196	0.030
We-words	19.08	26.35	0.000
I-words	119.50	130.36	0.040
Amy	2.65	7.19	0.002
Bernadette	3.62	5.48	0.136
Emily	0	0.55	0.001
Howard	14.19	12.88	0.502
Leonard	24.46	20.85	0.157
Leslie Winkle	0.23	0.28	0.898
Penny	17.04	17.06	0.992
Raj	9.31	9.56	0.866
Sheldon	28.50	25.25	0.205
Stuart	0.42	1.52	0.001

If we set a 5% confidence level, we observe a significant difference between the Top 10% and the bottom 90% (rejection of the null hypothesis in the t-test) for the following features:

- Male percentage;
- Complexity;
- HHI;
- We-words;
- I-words;
- Amy;
- Emily;
- Stuart.

The list confirms what we found with Cohen’s *f*^2^ analysis, with the addition of two more characters (Amy and Emily) and the I-words predictor.

Similarly, in Table 6, we report the slopes for the multilevel model when the dependent variable is the number of voters, which is an indicator of popularity. Again, the full model inclusive of gender is much more accurate than what we get by removing the gender information, but even without gender information the full model retains a 27.4% reduction in the variance with respect Model 1 and a 4.2% reduction with respect to the male percentage-based single predictor model.

**Table 6:** Slopes and variances in the multilevel model for voters (†*p* < 0.001; ⊗*p* < 0.01; *p < 0.05).

Predictor	Model									
	1	2	3	4	5	6	7	8	9	10
Episode		−16.277^†^							−16.743^†^	−17.813^†^
Male percent.			−13673^†^						−13430^†^	
Complexity				8.195^†^					10.584^†^	11.57^†^
Sentiment				-2131					180.7	384
No. of words					0.07				−0.331*	−0.346*
I-words						-0.485			0.429	0.03
We-words						-1.537			-2.085	-0.803
HHI							529		-866	-863
Amy								2.454	-5.045	-0.297
Bernadette								0.257	2.277	-1.192
Emily								-12.593	-4.506	-8.422
Howard								-0.072	-1.946	-2.632
Leonard								4.248*	1.214	0.676
Leslie								-5.055	-12.967	-16.853
Penny								3.902	-0.335	0.402
Raj								-3.462	1.039	-2.480
Sheldon								2.87	-0.206	-0.65
Stuart								-7.68	-9.256	-10.0
Intercept	2008^†^	2203^†^	13378^†^	2963^†^	1898^†^	2109^†^	1901^†^	1808^†^	13723^†^	2515^⊗^
L2 Variance	64304	57977	32462	43419	61448	59905	56154	44691	23582	52497
L1 Variance	78890	66730	59706	73750	78922	78804	78765	74747	41077	57204

**Fig 8:**
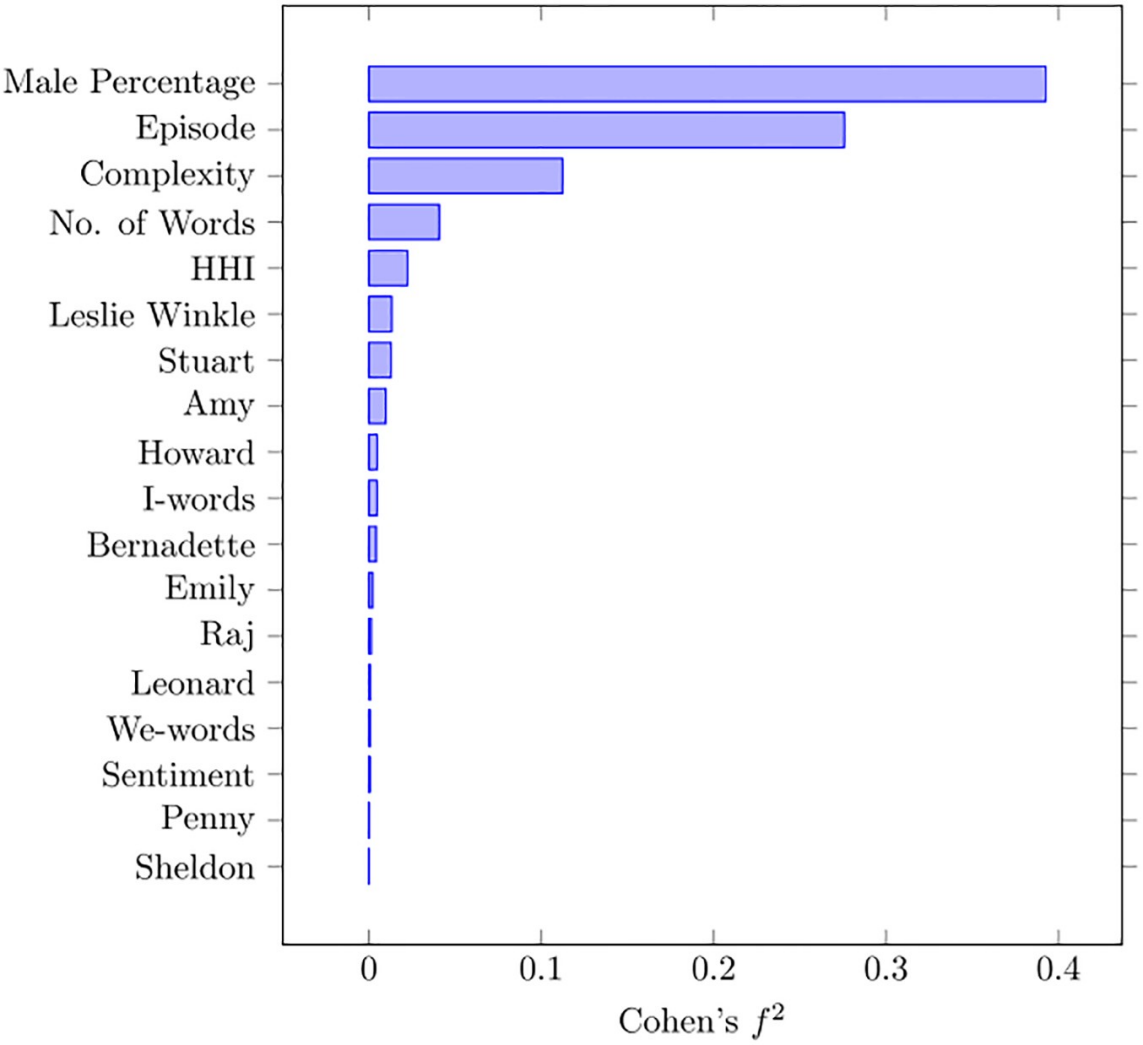
Cohen’s *f*^2^ for voters.

We can carry out a similar effect size analysis for Voters as we did for the Rating. In Fig 8, we see that the predictors with the largest effect size are:

- Male percentage;
- Episode number;
- Complexity;
- Number of words.

Both for Rating and Voters the percentage of male users is one of the most important factors. A higher language complexity also seems to positively affect episode popularity. As to the episode number, we have already noted that the number of voters declines with the age of the series, as shown in Fig 3, so that the popularity of the series has actually decreased over the seasons.

Since the *f*^2^ analysis allows us to identify the most relevant factors for popularity, we can build a parsimonious model out of them. We report the coefficients of the model in Table 7. If we look at the L1 variance (i.e., the level of episodes), we see that the parsimonious model, though being slightly worse than the full model (exhibiting 8.3% more variance), it achieves a far lower variance than the best of the single-predictor models (34.2% less variance).

**Table 7:** Slopes and variances in the parsimonious model for voters (†*p* < 0.001; ⊗*p* < 0.01).

Predictor	Weight
Episode number	−15.36^†^
Male percentage	−12441^†^
Complexity	9.12^†^
No. of words	−0.338^⊗^
Intercept	12823^†^
L2 Variance	27123
L1 Variance	44479

Finally, we perform a collinearity analysis through the Variance Inflation Factor (VIF), as defined in [43]. A commonly used rule of thumb is that any VIF of 10 or more provides evidence of serious multicollinearity (see again [43]). In our case the values we obtain are all between 1 and 2, therefore way below the above mentioned threshold. We can conclude the there is no significant collinearity.

## Conclusions

As a first step towards the identification of predictors for the performance of a TV series, we have considered the case of the Big Bang Theory, a TV series that has received a number of awards.

We have focussed on extracting predictors of performance from the TV series script itself, by applying text mining and social network analysis.

By using correlation and regression analysis, we have identified the most relevant predictors of popularity and appreciation. In particular, the presence of male viewers and the complexity of the language affect both popularity and appreciation: the influence of male viewers is negative, while the complexity of the language has a positive influence (this may be expected, given the social and working context of the characters). In addition, the presence of dominant characters, contributing most to the dialogues, is a relevant factor for appreciation. The popularity appears to decrease as the series goes on. Since gender cannot be used as a predictor, we have also considered a model where the gender is absent. Its describing capability was however worse, so that we can conclude that the gender of voters has anyway a relevant role in the rating scored by the series.

This contribution may provide an interesting support to orientate the design of a TV series by identifying the factors that most contribute to its performance and suggesting additional tools to measure those factors.

## Supporting information

S1 Table Dialogue list. The table reports the speaker (first column) and the recipient (second column) of each dialogue line for all episodes (season and episode are reported in the third column).
(CSV)

## References

[1] Fu P, Zhu A, Fang Q, Wang X. Modeling Periodic Impulsive Effects on Online TV Series Diffusion. PloS one. 2016;11(9):e0163432 10.1371/journal.pone.0163432 27669520 PMC5036804

[2] Bhave A, Kulkarni H, Biramane V, Kosamkar P. Role of different factors in predicting movie success. In: Pervasive Computing (ICPC), 2015 International Conference on. IEEE; 2015. p. 1–4.

[3] Delmestri G, Montanari F, Usai A. Reputation and strength of ties in predicting commercial success and artistic merit of independents in the Italian feature film industry. Journal of Management Studies. 2005;42(5):975–1002. 10.1111/j.1467-6486.2005.00529.x

[4] Mestyán M, Yasseri T, Kertész J. Early prediction of movie box office success based on Wikipedia activity big data. PloS one. 2013;8(8):e71226 10.1371/journal.pone.0071226 23990938 PMC3749192

[5] Krauss J, Nann S, Simon D, Gloor PA, Fischbach K. Predicting Movie Success and Academy Awards through Sentiment and Social Network Analysis. In: ECIS; 2008. p. 2026–2037.

[6] Austin BA. The influence of the MPAA’s film-rating system on motion picture attendance: A pilot study. The Journal of Psychology. 1980;106(1):91–99. 10.1080/00223980.1980.9915174

[7] Chang BH, Ki EJ. Devising a practical model for predicting theatrical movie success: Focusing on the experience good property. Journal of Media Economics. 2005;18(4):247–269. 10.1207/s15327736me1804_2

[8] Jain V. Prediction of movie success using sentiment analysis of tweets. The International Journal of Soft Computing and Software Engineering. 2013;3(3):308–313.

[9] Sadikov E, Parameswaran AG, Venetis P. Blogs as Predictors of Movie Success. In: ICWSM; 2009.

[10] Sharda R, Delen D. Predicting box-office success of motion pictures with neural networks. Expert Systems with Applications. 2006;30(2):243–254. 10.1016/j.eswa.2005.07.018

[11] Kennedy RE. Strategy fads and competitive convergence: An empirical test for herd behavior in prime-time television programming. The Journal of Industrial Economics. 2002;50(1):57–84. 10.1111/1467-6451.00168

[12] Barroso A, Giarratana MS, Reis S, Sorenson O. Crowding, satiation, and saturation: The days of television series’ lives. Strategic Management Journal. 2016;37(3):565–585. 10.1002/smj.2345

[13] Khessina OM, Reis S. The limits of reflected glory: The beneficial and harmful effects of product name similarity in the US network TV program industry, 1944–2003. Organization Science. 2016;27(2):411–427. 10.1287/orsc.2015.1036

[14] Wei-Skillern J, Marciano S. Primer on the US television industry. Harvard Business School Background Note 308-128, Boston; 2008.

[15] Eliashberg J, Shugan SM. Film critics: Influencers or predictors? Journal of marketing. 1997;61(2):68–78. 10.2307/1251831

[16] Hur M, Kang P, Cho S. Box-office forecasting based on sentiments of movie reviews and Independent subspace method. Information Sciences. 2016;372:608–624. 10.1016/j.ins.2016.08.027

[17] Silverman BW. Density estimation for statistics and data analysis. Routledge; 2018.

[18] Abrahamsson H, Nordmark M. Program Popularity and Viewer Behaviour in a Large TV-on-demand System. In: Proceedings of the 2012 Internet Measurement Conference. IMC’12. New York, NY, USA: ACM; 2012. p. 199–210. Available from: http://doi.acm.org/10.1145/2398776.2398798.

[19] Fronzetti Colladon A, Vagaggini F. Robustness and stability of enterprise intranet social networks: The impact of moderators. Information Processing & Management. 2017;53(6):1287–1298. 10.1016/j.ipm.2017.07.001

[20] Antonacci G, Fronzetti Colladon A, Stefanini A, Gloor PA. It is Rotating Leaders Who Build the Swarm: Social Network Determinants of Growth for Healthcare Virtual Communities of Practice. Journal of Knowledge Management. 2017;21(5):1218–1239.

[21] Chen X, Meurers D. Characterizing Text Difficulty with Word Frequencies. In: Proceedings of the 11th Workshop on Innovative Use of NLP for Building Educational Applications; 2016. p. 84–94.

[22] Baixeries J, Elvevåg B, Ferrer-i Cancho R. The evolution of the exponent of Zipf’s law in language ontogeny. PloS one. 2013;8(3):e53227 10.1371/journal.pone.0053227 23516390 PMC3596411

[23] Zipf GK. Human behavior and the principle of least effort: An introduction to human ecology. Ravenio Books; 2016.

[24] Piantadosi ST. Zipf’s word frequency law in natural language: A critical review and future directions. Psychonomic bulletin & review. 2014;21(5):1112–1130. 10.3758/s13423-014-0585-6 24664880 PMC4176592

[25] Naldi M. Approximation of the truncated Zeta distribution and Zipf’s law. arXiv preprint series, arXiv:151101480. 2015.

[26] Gloor PA. Sociometrics and Human Relationships: Analyzing Social Networks to Manage Brands, Predict Trends, and Improve Organizational Performance. London, UK: Emerald Publishing Limited; 2017.

[27] Gloor PA, Zhao Y. Tecflow-a temporal communication flow visualizer for social networks analysis. In: ACM CSCW Workshop on Social Networks. vol. 6; 2004.

[28] Amancio DR, Comin CH, Casanova D, Travieso G, Bruno OM, Rodrigues FA, et al A systematic comparison of supervised classifiers. PloS one. 2014;9(4):e94137 10.1371/journal.pone.0094137 24763312 PMC3998948

[29] Gloor PA, Krauss J, Nann S, Fischbach K, Schoder D. Web Science 2.0: Identifying Trends through Semantic Social Network Analysis. In: 2009 International Conference on Computational Science and Engineering. Vancouver, Canada: IEEE; 2009. p. 215–222. Available from: http://ieeexplore.ieee.org/document/5284145/.

[30] Brönnimann L. Multilanguage sentiment-analysis of Twitter data on the example of Swiss politicians; 2013.

[31] Brönnimann L. Analyse der Verbreitung von Innovationen in sozialen Netzwerken [M.Sc. Thesis]. University of Applied Sciences Northwestern Switzerland; 2014. Available from: http://www.twitterpolitiker.ch/documents/Master_Thesis_Lucas_Broennimann.pdf.

[32] Pennebaker JW. The Secret Life of Pronouns: What our Words Say about us. New York, NY: Bloomsbury Press; 2011.

[33] Pennebaker JW, Chung CK, Frazee J, Lavergne GM, Beaver DI. When small words foretell academic success: The case of college admissions essays. PloS one. 2014;9(12):e115844 10.1371/journal.pone.0115844 25551217 PMC4281205

[34] Chung CK, Pennebaker JW. Using computerized text analysis to track social processes Handbook of language and social psychology New York: Oxford 2013; p. 12.

[35] Scholand A, Tausczik Y, Pennebaker J. Linguistic analysis of workplace computer-mediated communication. Proceedings of Computer Supported Cooperative Work 2010. 2010.

[36] Hancock JT, Beaver DI, Chung CK, Frazee J, Pennebaker JW, Graesser A, et al Social language processing: A framework for analyzing the communication of terrorists and authoritarian regimes. Behavioral Sciences of Terrorism and Political Aggression. 2010;2(2):108–132. 10.1080/19434471003597415

[37] Rhoades SA. The Herfindahl-Hirschman Index. Fed Res Bull. 1993;79:188.

[38] Naldi M. Concentration indices and Zipf’s law. Economics Letters. 2003;78(3):329–334. 10.1016/S0165-1765(02)00251-3

[39] Khan HH, Ahmad RB, Gee CS. Market structure, financial dependence and industrial growth: Evidence from the banking industry in emerging Asian economies. PloS one. 2016;11(8):e0160452 10.1371/journal.pone.0160452 27490847 PMC4973879

[40] Sheskin DJ. Handbook of parametric and nonparametric statistical procedures. crc Press; 2003.

[41] Gelman A, Hill J. Data analysis using regression and multilevel/hierarchical models. Cambridge university press; 2006.

[42] Brown MB, Forsythe AB. Robust tests for the equality of variances. Journal of the American Statistical Association. 1974;69(346):364–367. 10.1080/01621459.1974.10482955

[43] Cohen J, Cohen P, West SG, Aiken LS. Applied multiple regression/correlation analysis for the behavioral sciences. Routledge; 2013.

